# Role of Saroglitazar in Improving Transient Elastography Parameters in Significant and Advanced Metabolic Dysfunction-Associated Steatohepatitis

**DOI:** 10.7759/cureus.99044

**Published:** 2025-12-12

**Authors:** Manish C Kak

**Affiliations:** 1 Gastroenterology, Kak Liver and Gut Center, Nehru Nagar, Ghaziabad, IND

**Keywords:** controlled attenuation parameter, dyslipidemia, liver stiffness, masld, saroglitazar

## Abstract

Introduction: Metabolic dysfunction-associated steatohepatitis (MASH) contributes significantly to liver-related and cardiometabolic morbidity. Saroglitazar, a dual peroxisome proliferator-activated receptor (PPAR)-α/γ agonist, targets both hepatic and metabolic pathways. This study evaluated its real-world efficacy in improving liver stiffness, steatosis, hepatic transaminases, and lipid parameters.

Methods: This retrospective, single-center, observational study included 204 adults with metabolic dysfunction-associated steatotic liver disease (MASLD)/MASH treated with saroglitazar 4 mg once daily for 52 weeks. Patients were categorized as Significant Fibrosis (<14 kPa; n = 104) or Advanced Fibrosis (≥14 kPa; n = 100) based on baseline liver stiffness measurement (LSM). Changes in LSM, controlled attenuation parameter (CAP), alanine aminotransferase (ALT), aspartate aminotransferase (AST), total cholesterol, and low-density lipoprotein cholesterol (LDL-C) were analyzed at baseline, 24 weeks, and 52 weeks.

Results: In the Significant Fibrosis group, mean LSM reduced from 10.31 ± 2.01 to 6.27 ± 1.44 kPa (-39.1%, p < 0.001) and CAP from 295.82 ± 49.34 to 241.01 ± 63.61 dB/m (-18.4%). ALT and AST declined by 49.5% and 43.4%, respectively, with total cholesterol and LDL-C reductions of 17.6% and 25.9%. In the Advanced Fibrosis group, LSM decreased from 17.96 ± 1.85 to 13.83 ± 1.42 kPa (-23.0%) and CAP from 317.05 ± 61.28 to 272.36 ± 52.38 dB/m (-14.1%), accompanied by ALT and AST reductions of 46.6% and 45.1%, and total cholesterol and LDL-C reductions of 18.3% and 25.7% (p < 0.001 for all). Saroglitazar was well-tolerated without serious adverse events.

Conclusion: Saroglitazar 4 mg daily was associated with significant improvements in liver stiffness, steatosis, transaminases, and lipid parameters over 52 weeks. These findings support its hepatometabolic potential across both early and advanced MASLD/MASH stages in real-world practice.

## Introduction

Metabolic dysfunction-associated steatotic liver disease (MASLD), formerly known as non-alcoholic fatty liver disease (NAFLD), represents a significant and growing global health concern. The condition, characterized by hepatic steatosis and at least one cardiometabolic risk factor, is closely linked to the rising prevalence of obesity and type 2 diabetes mellitus (T2DM). In India, a recent systematic review and meta-analysis by Shalimar et al. (2022) found that approximately one in three adults and children is affected by MASLD. The pooled prevalence was estimated at 38.6% in adults and 35.4% in children. Despite multiple studies on MASLD prevalence in India, prior data had limitations such as small sample sizes, a focus on high-risk groups, and a lack of pan-India studies, leaving the exact disease burden unknown. This highlights a critical research gap and the need for new, real-world data [1].

The natural history of MASLD varies widely, with some patients remaining in early steatosis while others progress to metabolic dysfunction-associated steatohepatitis (MASH) and cirrhosis. Liver fibrosis is the strongest predictor of liver-related and cardiovascular outcomes in MASLD [2]. Early identification and risk stratification of MASLD patients are therefore critical for preventing disease progression and associated complications. Accurate determination of fibrosis severity, such as with the METAVIR (Meta-analysis of Histological Data in Viral Hepatitis) scoring system, is essential because patients with F3-F4 fibrosis have markedly worse long-term prognosis compared to those with F2 or lower [3].

Transient elastography (TE) is a widely used non-invasive method for fibrosis staging. A large meta-analysis demonstrated that TE provides high diagnostic accuracy for significant fibrosis, with a pooled sensitivity of 84% and specificity of 82% [4]. Another study showed that TE can diagnose cirrhosis with accuracy exceeding 90% across chronic liver disease etiologies [5]. However, evidence also indicates that TE cannot reliably differentiate between intermediate fibrosis stages (F2 vs. F3) because of overlapping liver stiffness values, despite strong performance in identifying F1 and F4 stages [6].

The primary therapeutic goal in MASLD is the reduction of hepatic inflammation and fibrosis due to their strong association with hepatic and cardiometabolic complications. Evidence shows that lifestyle modification alone produces only modest improvement in fibrosis and steatosis, demonstrating the need for effective pharmacologic therapy [7]. Additionally, fibrosis severity is directly correlated with progression to cirrhosis, liver-related events, and mortality, making it the key therapeutic target [8]. A recent global meta-analysis confirmed that MASLD significantly increases the risk of adverse clinical events, including liver failure, hepatocellular carcinoma, and cardiovascular mortality, with risk rising proportionally with increasing fibrosis stage [9].

Peroxisome proliferator-activated receptors (PPARs) are nuclear lipid-activated transcription factors that regulate the expression of genes to control lipid and lipoprotein metabolism, glucose homeostasis, and the inflammatory process [10]. Saroglitazar is a novel double PPAR-α/γ agonist, non-thiazolidinediones (TZD), and non-fibric corrosive derivative, with an overwhelming PPAR-α agonistic activity.

Evidence supporting saroglitazar in MASLD continues to expand. A prospective real-world study in NAFLD with diabetic dyslipidemia demonstrated that 24 weeks of saroglitazar 4 mg led to significant reductions in alanine aminotransferase (ALT), aspartate aminotransferase (AST), liver stiffness measurement (LSM), and controlled attenuation parameter (CAP), along with improvements in glycemic and lipid parameters, supporting its hepatometabolic mechanisms [11]. More recently, a randomized four‑arm Indian trial compared saroglitazar, vitamin E, their combination, and lifestyle control, finding that saroglitazar reduced ALT while the combination achieved the broadest benefits, including significant reductions in liver stiffness measure (primary surrogate of fibrosis) and CAP, with concurrent improvement in glycemic and lipid parameters, suggesting potential additive or synergistic effects of dual therapy on liver and metabolic endpoints.

The present study aimed to extend and validate these findings in a real-world Indian cohort by assessing the effectiveness and safety of saroglitazar using standardized elastography-based endpoints across both significant (F1-F3) and advanced (F4) fibrosis groups. The diagnosis of MASLD in this study required evidence of hepatic steatosis on TE-CAP together with at least one cardiometabolic risk factor as per the 2023 American Association for the Study of Liver Diseases (AASLD) guidance.

Accordingly, the objective of this study was to assess the effectiveness of saroglitazar 4 mg daily over 52 weeks in improving liver stiffness, steatosis, hepatic enzymes, and lipid parameters in adults with MASLD/MASH across both significant and advanced fibrosis categories.

## Materials and methods

This was a retrospective, single-center, observational, open-label, single-arm study evaluating the safety and efficacy of saroglitazar 4 mg once daily in adults with MASLD/MASH managed in a real‑world outpatient setting.

This study was conducted in accordance with the ethical standards of the Declaration of Helsinki (2013 revision). It was a retrospective analysis of anonymized clinical data collected as part of routine medical care. Formal institutional ethics committee approval was not obtained as the study did not involve any prospective intervention or patient-identifiable data and was therefore exempt from full ethical review according to national guidelines for biomedical research involving human participants (Indian Council of Medical Research, 2017). Patient confidentiality was strictly maintained throughout the study, and no identifiable personal information was used or disclosed.

Inclusion and exclusion criteria

The study included patients aged 18 years and above who had a diagnosis of MASLD and were initiated on saroglitazar 4 mg daily. The diagnosis of MASLD was based on the 2023 AASLD guidelines, requiring the presence of hepatic steatosis on TE, in the absence of significant alcohol consumption or other known causes of chronic liver disease.

Patients aged ≥18 years with MASLD based on AASLD 2023 criteria were included. Diagnostic entry criteria included hepatic steatosis on TE-CAP together with at least one metabolic risk factor, ALT >1.5 × the upper limit of normal (ULN), and either LSM ≥7.5 kPa or CAP >240 dB/m.

Patients were excluded if they met any of the following criteria: Significant alcohol consumption, defined as a regular intake of >210 g/week in males and >140 g/week in females; concomitant use of known steatogenic drugs (e.g., corticosteroids, tamoxifen, amiodarone, methotrexate, or other drugs on a pre-specified list); evidence of acute liver inflammation (e.g., recent viral hepatitis, drug-induced liver injury) that would confound the baseline ALT level; presence of other chronic liver diseases (e.g., chronic hepatitis B or C, Wilson’s disease) or signs of liver decompensation (e.g., ascites, encephalopathy); confounding factors that could influence TE values (e.g., liver congestion, ascites, liver tumors, biliary obstructions); use of TZD, vitamin E (≥800 IU/day), or saroglitazar in the six months prior to the study; pregnancy, lactation, or women of childbearing potential not using effective contraception.

Data collection and measurements

Data were collected retrospectively from patient medical records. A standardized data collection form was used to systematically record demographic details, medical history, comorbidities (T2DM, dyslipidemia, hypertension), and concurrent medications. Anthropometric measurements, including height and weight, were taken using a calibrated SECA stadiometer (SECA GmbH & Co. KG, Hamburg, Germany) and a SECA digital scale (SECA GmbH & Co. KG), respectively. All devices were calibrated weekly to ensure accuracy. Waist circumference was measured at the midpoint between the iliac crest and the lowest rib.

To assess liver fibrosis and steatosis, the Echosens Transient Elastography® 530 Compact machine (Echosens, Paris, France) was used by an Echosens-certified technician. The M-probe was used for BMI <30 kg/m^2^ and the XL-probe for BMI ≥30 kg/m^2^. Reliability criteria included ≥10 valid measurements and an interquartile range (IQR)/median ratio ≤30%. For a subset of 20 randomly selected patients, two certified technicians independently performed the TE to assess inter-observer agreement.

Outcomes and classification

The primary outcome measures were the changes in liver stiffness (LSM) and steatosis (CAP) values from baseline to 12 months after starting saroglitazar therapy. Secondary outcomes included changes in ALT, AST, total cholesterol, and low-density lipoprotein cholesterol (LDL-C). For analysis, patients were classified into two groups based on their baseline LSM, which aligns with the accepted Indian National Association for the Study of the Liver (INASL) definitions for advanced liver disease: Significant to Advanced Fibrosis: LSM <14 kPa (F0-F3) and Cirrhosis: LSM ≥14 kPa (F4). This classification was chosen to evaluate the efficacy of saroglitazar in patients with late-stage fibrosis, which is a key predictor of clinical outcomes.

Standard of care

Although the study’s retrospective nature did not allow structured documentation of lifestyle adherence, all patients routinely received dietary and physical activity counselling as part of standard care, including recommendations for a Mediterranean or low-carbohydrate diet, a target of 150 minutes per week of moderate-intensity exercise in addition to saroglitazar 4 mg once daily. Diabetes and dyslipidemia were managed according to standard-of-care guidelines. Potential confounding variables, including changes in antidiabetic or lipid-lowering therapy, weight fluctuations, and glycemic control, were reviewed from follow-up records; however, residual confounding could not be fully excluded and is acknowledged as a limitation.

Statistical analysis

The statistical analysis was performed using IBM SPSS Statistics for Windows, Version 22 (Released 2013; IBM Corp., Armonk, New York, United States). Continuous variables were presented as mean ± standard deviation (SD) or median and IQR, as appropriate. Categorical variables were expressed as frequencies and percentages.

Normality of data distribution was assessed using the Shapiro-Wilk test. The differences in liver stiffness and steatosis measurements between baseline and 12 months were analyzed using a paired t-test for normally distributed data or the Wilcoxon signed-rank test for non-normally distributed data. For each comparison, both the test statistic (t-value) and the corresponding two-tailed p-value were calculated and reported. A two-tailed p-value of <0.05 was considered statistically significant.

Missing data were handled using the Last Observation Carried Forward (LOCF) method, and a complete-case sensitivity analysis was conducted to assess robustness. No patient was lost to follow-up for 52-week elastography assessments due to the structured and continuous care model of the center. A sensitivity analysis was also performed using a complete case analysis to ensure the robustness of the findings.

Inter-observer agreement for TE measurements was assessed using the intraclass correlation coefficient (ICC) on a subset of 20 patients. An ICC value of >0.85 was considered excellent agreement. Patient safety was assessed at each follow-up visit through a detailed history of potential side effects and clinical examination.

## Results

A total of 276 individuals were screened for the study. After excluding 72 patients who met one or more of the outlined exclusion criteria, 204 patients were included in the protocol analysis. Baseline investigations of all 204 patients were conducted. Table 1 summarizes the demographic and clinical parameters at baseline.

**Table 1:** Demographic profile of patients (n = 204). Data are expressed as mean ± SD or number (percentage).

Parameter	Observation
Age (years)	51.01 ± 11.29
Weight (kg)	76.01 ± 9.04
BMI (Kg/m^2^)	29.4 ± 5.07
Male, n (%)	114 (56.16)
Diabetic, n (%)	96 (47.06)
Hypertensive, n (%)	88 (43.14)

All 204 patients received saroglitazar 4 mg once daily and completed both 24-week and 52-week evaluations, with no loss to follow-up owing to structured continuity of care at the center.

Of the 204 patients, 104 (50.98%) were classified as having Significant Fibrosis (LSM <14 kPa) and 100 (49.02%) as having Advanced Fibrosis (LSM ≥14 kPa) based on baseline TE. The baseline elastography and biochemical characteristics of the two groups are presented in Table 2.

**Table 2:** Baseline characteristics of the two groups (Significant and Advanced Fibrosis). Data are expressed as mean ± SD or number (percentage). ALT: alanine aminotransferase; AST: aspartate aminotransferase; LDL-C: low-density lipoprotein cholesterol

Parameter	Significant Fibrosis (liver stiffness measure <14 kPa)	Advanced Fibrosis (liver stiffness measure >14 kPa)
Total subjects, n	104	100
Mean age (years)	49.16 ± 11.09	52.84 ± 11.21
Male, n (%)	58 (55.77)	58 (58)
Mean liver stiffness measure (kPa)	10.31 ± 2.01	17.96 ± 1.85
Mean controlled attenuation parameter (dB/m)	295.08 ± 49.34	317.15 ± 61.28
ALT (U/L)	76.4 ± 28.2	88.6 ± 31.7
AST (U/L)	65.2 ± 24.9	79.8 ± 26.3
Total cholesterol (mg/dL)	214.5 ± 39.8	221.7 ± 41.6
LDL-C (mg/dL)	132.9 ± 33.2	137.4 ± 35.1

**Figure 1:**
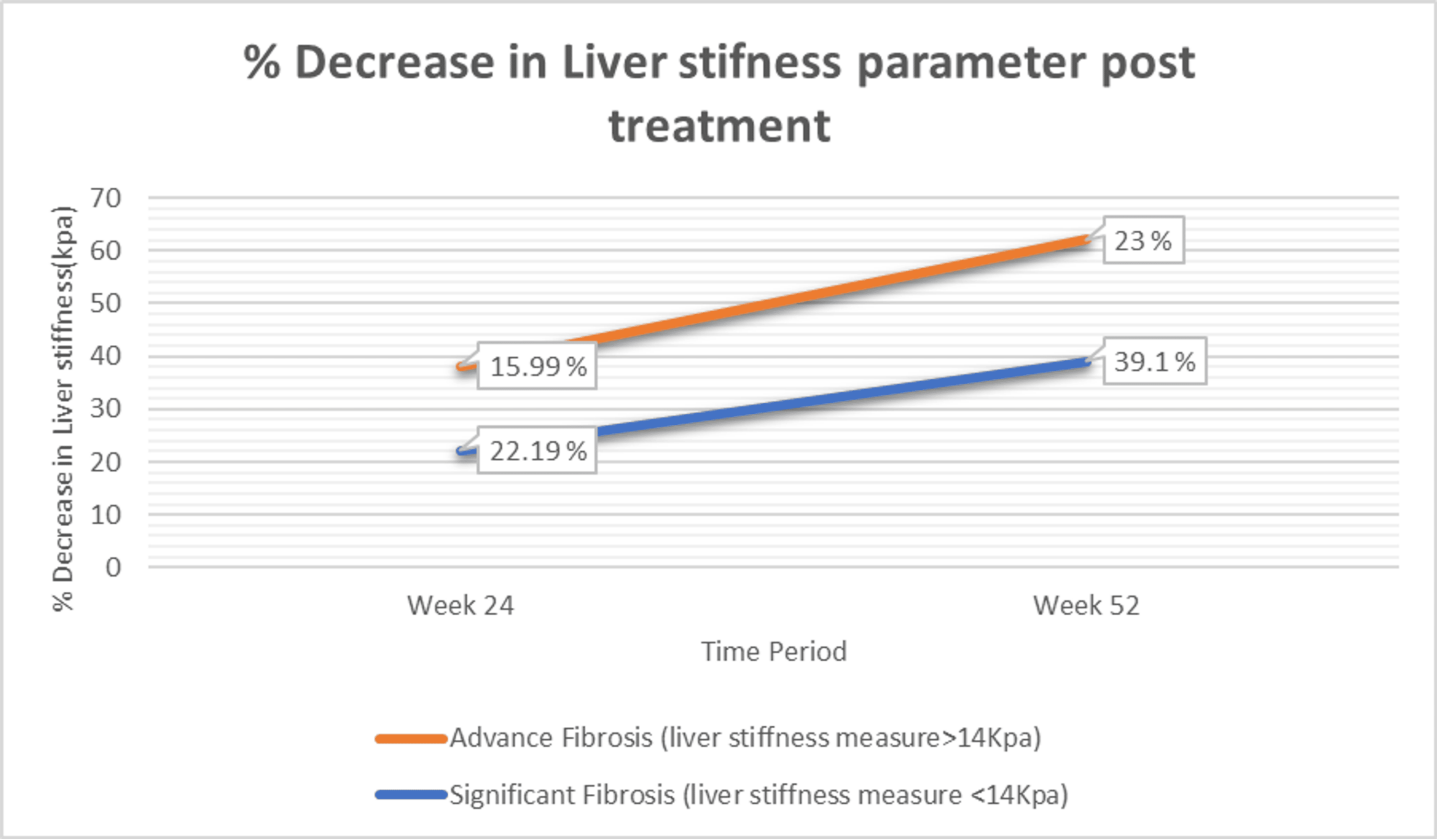
Percentage decrease in liver stiffness (transient elastography) following treatment with saroglitazar.

After treatment with saroglitazar 4 mg once daily for 24 and 52 weeks, patients with Significant Fibrosis (n = 104; baseline liver stiffness <14 kPa) demonstrated substantial improvements. The mean LSM decreased from 10.31 ± 2.01 kPa at baseline to 8.02 ± 1.99 kPa at 24 weeks, representing a significant 2.28-point (22.19%) reduction (p < 0.001). By 52 weeks, LSM further declined to 6.27 ± 1.44 kPa, corresponding to a 4.03-point (39.10%) reduction from baseline (t = 18.42; p < 0.001), as shown in Figure 1.

**Figure 2:**
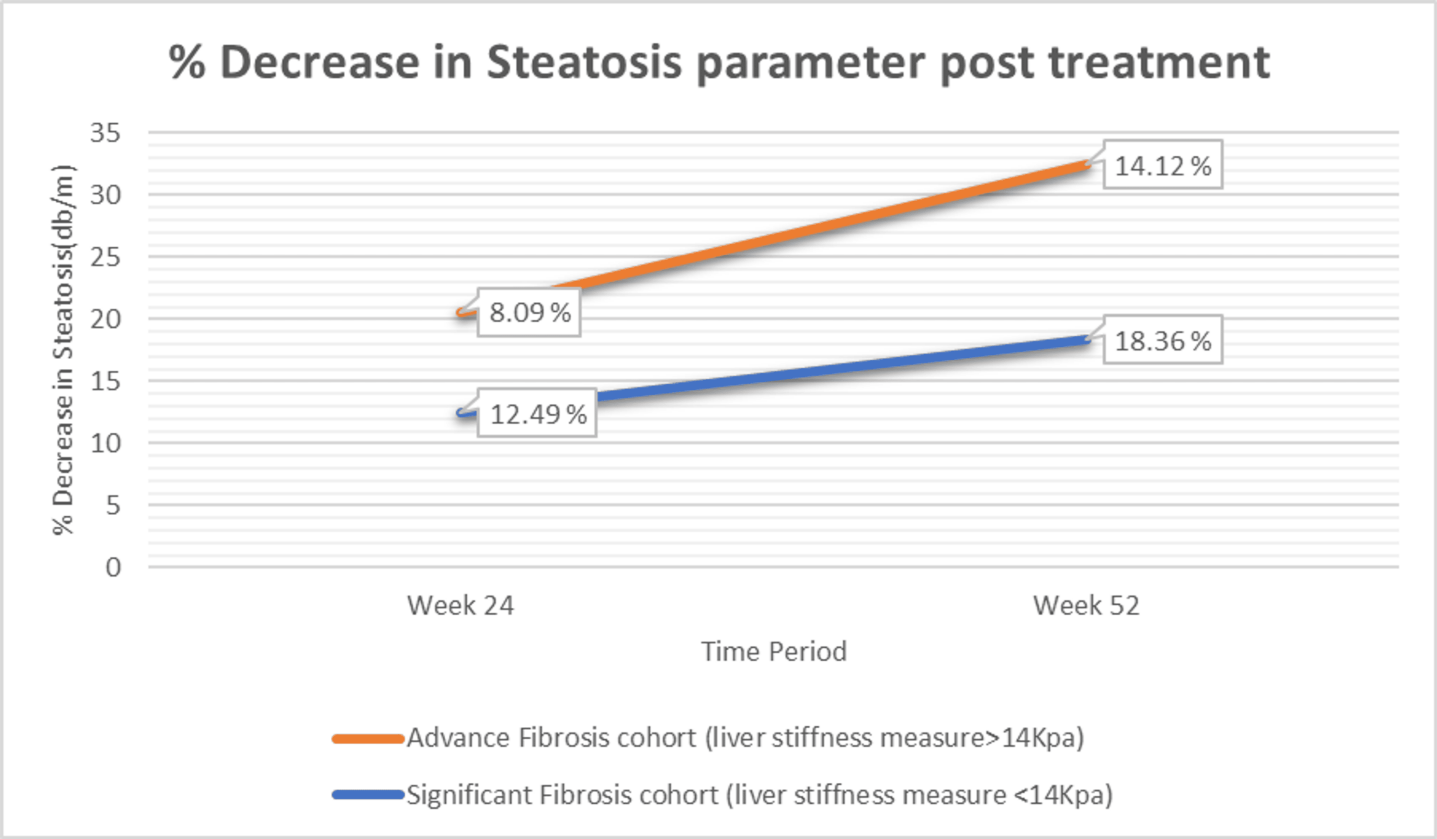
Percentage decrease in steatosis (transient elastography) following treatment with saroglitazar.

The CAP value decreased from 295.82 ± 49.34 dB/m at baseline to 258.85 ± 52.45 dB/m at 24 weeks, representing a significant 36.97-point (12.49%) reduction (p < 0.001). By 52 weeks, CAP further declined to 241.01 ± 63.61 dB/m, corresponding to a 54.32-point (18.36%) reduction from baseline (t = 10.76; p < 0.001), as shown in Table 3 and Figure 2.

**Table 3:** Results following therapy (Significant and Advanced Fibrosis groups). Data are expressed as mean ± SD. ALT: alanine aminotransferase; AST: aspartate aminotransferase; LDL-C: low-density lipoprotein cholesterol

Parameter	Significant Fibrosis (liver stiffness measure <14 kPa)					Advanced Fibrosis (liver stiffness measure >14 kPa)				
	Baseline	Week 24	Week 52	t-value (Week 52)	p-value (Week 52)	Baseline	Week 24	Week 52	t-value (Week 52)	p-value (Week 52)
Mean liver stiffness measure (kPa)	10.31 ± 2.01	8.02 ± 1.99	6.27 ± 1.44	t = 18.42	<0.001	17.96 ± 1.85	15.09 ± 1.55	13.83 ± 1.42	t = 17.55	<0.001
Mean controlled attenuation parameter (dB/m)	295.82 ± 49.34	258.85 ± 52.45	241.01 ± 63.61	t = 10.76	<0.001	317.05 ± 61.28	291.47 ± 56.26	272.36 ± 52.38	t = 8.92	<0.001
ALT (U/L)	76.4 ± 28.2	52.7 ± 23.9	38.6 ± 20.1	t = 14.88	<0.001	88.6 ± 31.7	64.8 ± 26.2	47.3 ± 21.7	t = 13.74	<0.001
AST (U/L)	65.2 ± 24.9	47.6 ± 21.4	36.9 ± 18.6	t = 12.96	<0.001	79.8 ± 26.3	59.3 ± 22.5	43.8 ± 19.4	t = 12.55	<0.001
Total cholesterol (mg/dL)	214.5 ± 39.8	189.3 ± 34.7	176.8 ± 32.4	t = 11.41	<0.001	221.7 ± 41.6	194.6 ± 37.2	181.2 ± 34.1	t = 10.22	<0.001
LDL-C (mg/dL)	132.9 ± 33.2	110.8 ± 29.5	98.4 ± 26.3	t = 12.38	<0.001	137.4 ± 35.1	114.6 ± 30.1	102.1 ± 27.8	t = 11.65	<0.001

Mean ALT levels decreased from 76.4 ± 28.2 U/L at baseline to 52.7 ± 23.9 U/L at 24 weeks (31.0% reduction) and to 38.6 ± 20.1 U/L at 52 weeks (49.5% reduction, t = 14.88, p < 0.001). Mean AST levels declined from 65.2 ± 24.9 U/L to 47.6 ± 21.4 U/L at 24 weeks (27.0% reduction) and to 36.9 ± 18.6 U/L at 52 weeks (43.4% reduction, t = 12.96, p < 0.001). Parallel improvements were observed in lipid parameters, with total cholesterol decreasing from 214.5 ± 39.8 mg/dL to 189.3 ± 34.7 mg/dL (11.7% reduction) and 176.8 ± 32.4 mg/dL (17.6% reduction, t = 11.41) at 24 and 52 weeks, respectively. Mean LDL-C levels declined from 132.9 ± 33.2 mg/dL at baseline to 110.8 ± 29.5 mg/dL (16.6% reduction) and 98.4 ± 26.3 mg/dL (25.9% reduction, t = 12.38) by 24 and 52 weeks (p < 0.001 for all comparisons) as shown in Table 3.

Of 98 Advance fibrosis patients (liver stiffness measure >14 KPA) mean liver stiffness measure value decreased from 17.96 ± 1.85 to 15.09 ± 1.55 at 24 weeks, representing a significant 2.87-point (15.99%) (p <0.001) reduction and at 52 weeks to 13.83 ± 1.42, representing a significant 4.13 point (23%) (t = 17.55, P value <0.001) reduction from baseline, shown in (Figure 1).

The CAP value decreased from 317.05 ± 61.28 to 291.47 ± 56.26 at 24 weeks, representing a significant 25.68-point (8.09%) (P value <0.001) reduction and at 52 weeks to 272.36 ± 52.38, representing a significant 44.7-point (14.12%) (t = 8.92, P value <0.001) reduction from baseline, shown in Table 3 and Figure 2.

Mean ALT decreased from 88.6 ± 31.7 U/L at baseline to 64.8 ± 26.2 U/L at 24 weeks (26.8% reduction) and 47.3 ± 21.7 U/L at 52 weeks (46.6% reduction, t = 13.74, p < 0.001). Mean AST reduced from 79.8 ± 26.3 U/L to 59.3 ± 22.5 U/L (25.7% reduction) and further to 43.8 ± 19.4 U/L (45.1% reduction, t = 12.55, p < 0.001). Concurrently, total cholesterol fell from 221.7 ± 41.6 mg/dL to 194.6 ± 37.2 mg/dL (12.2% reduction) and 181.2 ± 34.1 mg/dL (18.3% reduction, t = 10.22,) at 24 and 52 weeks, respectively, while LDL-C declined from 137.4 ± 35.1 mg/dL to 114.6 ± 30.1 mg/dL (16.6% reduction) and 102.1 ± 27.8 mg/dL (25.7% reduction, t = 11.65, p < 0.001) as shown in Table 3.

It was noticed that saroglitazar, at a once-daily dose of 4 mg, was safe and well-tolerated because there were no serious adverse events associated with the medication that required treatment cessation. All 204 patients completed the follow-up for 12 months and were included in the post-treatment data analysis.

## Discussion

This real-world, retrospective observational study evaluated the effectiveness and safety of saroglitazar 4 mg once daily in adults with MASLD/MASH across both significant (F1-F3) and advanced (F4) fibrosis stages as assessed by TE. The findings demonstrated consistent and statistically significant reductions in LSM, CAP, hepatic transaminases, and lipid parameters over 52 weeks. These improvements were observed in both early and advanced fibrosis groups, suggesting a favorable hepatometabolic response to therapy across a broad disease spectrum.

The improvements in LSM and CAP observed in this study are consistent with previous Indian real-world data. Chaudhuri et al. reported significant reductions in elastography parameters, including among compensated cirrhotic patients, following saroglitazar therapy [12]. Similarly, the present study demonstrated a 39.1% reduction in LSM in the Significant Fibrosis group and a 23.0% reduction in the Advanced Fibrosis group at 52 weeks, findings that align closely with these earlier observations. The inclusion of a sizeable proportion of patients with baseline LSM ≥14 kPa further highlights the potential applicability of saroglitazar in patients with advanced fibrosis, a subgroup often underrepresented in clinical trials.

Prior research by Padole et al. suggested that elastography improvements may be modulated by weight reduction, with patients achieving greater than 5% weight loss demonstrating the most robust reductions in LSM and CAP [13]. In contrast, the improvements documented in the present cohort were not directly attributable to weight change, as detailed weight data and structured weight-loss interventions were not available due to the retrospective design. This observation raises the possibility that saroglitazar may contribute to fibrosis and steatosis reduction through mechanisms independent of weight loss, potentially reflecting its dual PPAR-α/γ pharmacologic activity.

The significant reductions in ALT and AST in both fibrosis groups observed in this study further support the hepatocellular benefits of saroglitazar. These findings are consistent with previously published real-world studies demonstrating improvements in biochemical markers of hepatic inflammation following therapy [11]. Importantly, reductions in total cholesterol and LDL-C were also noted, underscoring the drug’s beneficial impact on dyslipidemia - an important metabolic comorbidity in MASLD/MASH.

Despite these encouraging observations, several limitations must be acknowledged. As a retrospective study without a comparator arm, causal inferences cannot be made, and the observed improvements may have been influenced to some extent by unmeasured confounding variables, including variations in adherence to diet, exercise, or concomitant medications. Although lifestyle counseling was routinely provided as part of standard clinical practice, detailed documentation of adherence, caloric intake, or exercise frequency was not consistently available. Similarly, changes in glycemic control or adjustments in antidiabetic or lipid-lowering therapy may have contributed to the observed metabolic improvements.

Reviewer concern regarding the absence of dropouts is understandable; however, all patients remained under continuous follow-up within a structured single-center clinical program, facilitating complete dataset retention. While this high retention strengthens internal completeness of the dataset, it may limit generalizability to settings with less consistent follow-up.

Another important limitation involves the reliance on TE rather than histology. Although TE is widely validated for non-invasive fibrosis assessment, it has known limitations, particularly in distinguishing intermediate fibrosis stages (F2 vs. F3) and in populations with obesity or metabolic dysfunction. This limitation is acknowledged and highlights the need for future prospective studies incorporating histologic endpoints. Likewise, cardiovascular outcomes, which are increasingly recognized as the leading cause of mortality in MASLD, were not evaluated, and future long-term studies should integrate composite hepatic and cardiometabolic endpoints.

The retrospective design of this study, reliance on routinely collected clinical data, and potential for unmeasured confounding underscore the need for cautious interpretation. Nonetheless, the consistency, magnitude, and statistical significance of improvements across elastography and biochemical parameters suggest that saroglitazar may offer meaningful therapeutic benefits in MASLD/MASH, particularly in settings where real-world, non-invasive monitoring is central to patient management.

Overall, while the findings cannot establish causality, they contribute valuable real-world evidence supporting the hepatometabolic effects of saroglitazar and provide a foundation for future prospective, controlled, and histology-based investigations.

## Conclusions

In this real-world, single-center retrospective study, saroglitazar 4 mg once daily was associated with significant improvements in liver stiffness, hepatic steatosis, transaminases, and lipid parameters over a 52-week treatment period in patients with MASLD/MASH across both significant (F1-F3) and advanced (F4) fibrosis stages. The treatment was well-tolerated, and no serious adverse events were observed. Given the retrospective design and lack of a control group, these findings should be interpreted as descriptive rather than causal; however, the consistency and magnitude of improvements across multiple clinically relevant endpoints suggest a potential hepatometabolic benefit of saroglitazar in routine clinical practice. Future randomized, controlled, and prospective studies with histological endpoints and cardiovascular follow-up are warranted to validate these observations and determine the long-term therapeutic role of saroglitazar in MASLD/MASH.
